# Rift Valley Fever in Humans and Animals in Mayotte, an Endemic Situation?

**DOI:** 10.1371/journal.pone.0074192

**Published:** 2013-09-30

**Authors:** Tinne Lernout, Eric Cardinale, Maël Jego, Philippe Desprès, Louis Collet, Betty Zumbo, Emmanuel Tillard, Sébastien Girard, Laurent Filleul

**Affiliations:** 1 Regional office (Cire) of the French Institute for Public Health Surveillance (Institut de veille sanitaire), Mamoudzou, Mayotte, France; 2 Emerging and Exotic Animal Disease Control Joint Research Unit (UMR CMAEE), French Agricultural Research Centre for International Development (CIRAD), Montpellier, France; 3 Centre for Research and Surveillance of Emerging Diseases in the Indian Ocean (CRVOI), Cyclotron Réunion Indian Ocean Platform (Cyroi), Saint Denis, La Réunion, France; 4 Interactions Moléculaires Flavivirus-Hôtes Unit & WHO Collaborating Centre for Reference and Research on Arboviruses and Viral Haemorrhagic fevers, Institut Pasteur, Paris, France; 5 Laboratory of the Hospital Centre of Mayotte (CHM), Mamoudzou, Mayotte, France; 6 Vector control unit, Public Health Authority (Agence de santé océan Indien), Mamoudzou, Mayotte, France; 7 Tropical and Mediterranean Animal Production Systems Joint Research Unit (UMR SELMET), French Agricultural Research Centre for International Development (CIRAD), Montpellier, France; George Mason University, United States of America

## Abstract

Retrospective studies and surveillance on humans and animals revealed that Rift Valley Fever virus (RVFV) has been circulating on Mayotte for at least several years. A study was conducted in 2011 to estimate the seroprevalence of RVF in humans and in animals and to identify associated risk factors. Using a multistage cluster sampling method, 1420 individuals were enrolled in the human study, including 337 children aged 5 to 14 years. For the animal study, 198 seronegative ruminants from 33 randomly selected sentinel ruminant herds were followed up for more than one year. In both studies, information on environment and risk factors was collected through a standardized questionnaire. The overall weighted seroprevalence of RVFV antibodies in the general population aged ≥5 years was 3.5% (95% CI 2.6–4.8). The overall seroprevalence of RVFV antibodies in the ruminant population was 25.3% (95% CI 19.8–32.2). Age (≥15), gender (men), place of birth on the Comoros, living in Mayotte since less than 5 years, low educational level, farming and living close to a water source were significantly associated with RVFV seropositivity in humans. Major risk factors for RFV infection in animals were the proximity of the farm to a water point, previous two-month rainfall and absence of abortions disposal. Although resulting in few clinical cases in humans and in animals, RVFV has been circulating actively on the island of Mayotte, in a context of regular import of the virus from nearby countries through illegal animal movements, the presence of susceptible animals and a favorable environment for mosquito vectors to maintain virus transmission locally. Humans and animals share the same ways of RVFV transmission, with mosquitoes playing an important role. The studies emphasize the need for a one health approach in which humans and animals within their ecosystems are included.

## Introduction

Rift Valley Fever (RVF) is a mosquito-borne zoonosis that affects domestic animals and humans [1]. The RVF virus (RVFV) was first detected in Kenya [2] but later spread on the African continent and Yemen [1]. Among important RVF epizootics and epidemics reported, many occurred in East-African countries, geographically close to Mayotte. In 2006–2007, a large outbreak spread from Kenya to Tanzania [3] and Madagascar [4].

Humans are infected by RVFV through contact with blood or organs of infected animals, during slaughtering or when handling infected animals and contaminated meat. Transmission of the virus also results of bites from mosquitoes and possibly other bloodsucking vectors [1].

Following the identification of the first human case of RVF on Mayotte, imported from the Comoros Islands in 2007, retrospective studies were conducted on humans and animals and prospective surveillance set up [5]–[7]. Results revealed that RVF virus has been circulating on the island for at least several years, even before the detection of the first human case, without leading to detectable clinical cases in neither animals nor humans.

A study was conducted in 2011 to estimate the seroprevalence of Rift Valley Fever in the general population and in animals (ruminant population) in Mayotte, as well as to identify factors associated with human and animal RVF infection on the island.

## Materials and Methods

### Setting, design and population

Mayotte is a French overseas department located in the Indian Ocean, between the Eastern African coast and Madagascar. The island is very densely populated and has around 200,000 inhabitants of whom 53% are under 20 years of age, on a surface of 374 km^2^ [8]. Forty per cent of the population are foreigners, most of them illegal immigrants coming from the Comoros. Given the proximity of Mayotte and the Comoros, both part of the Comoros archipelago, travel (legal and illegal movements) between the islands is frequent. General hygiene and living conditions on Mayotte are poor.

The human serosurvey used a multistage cluster sampling method. First, 60 small geographical units (districts) were randomly selected among a total of 783, with proportionate probability to their size. In each district, a number of households were randomly selected to participate to the study, until inclusion of at least 24 individuals per district. Finally, in each household three persons were invited to participate by simple random sampling, including one child aged 5 to 14 years and two adults (≥15 years). If only 1 child and/or 2 adults were living in the house, all individuals were invited to participate. Up to two additional house visits were made at different times of the day and week to allow inclusion of absent members or households. The study objects were enrolled over a five week-period in March 2011, in accordance with written informed consent procedures.

**Figure 1:**
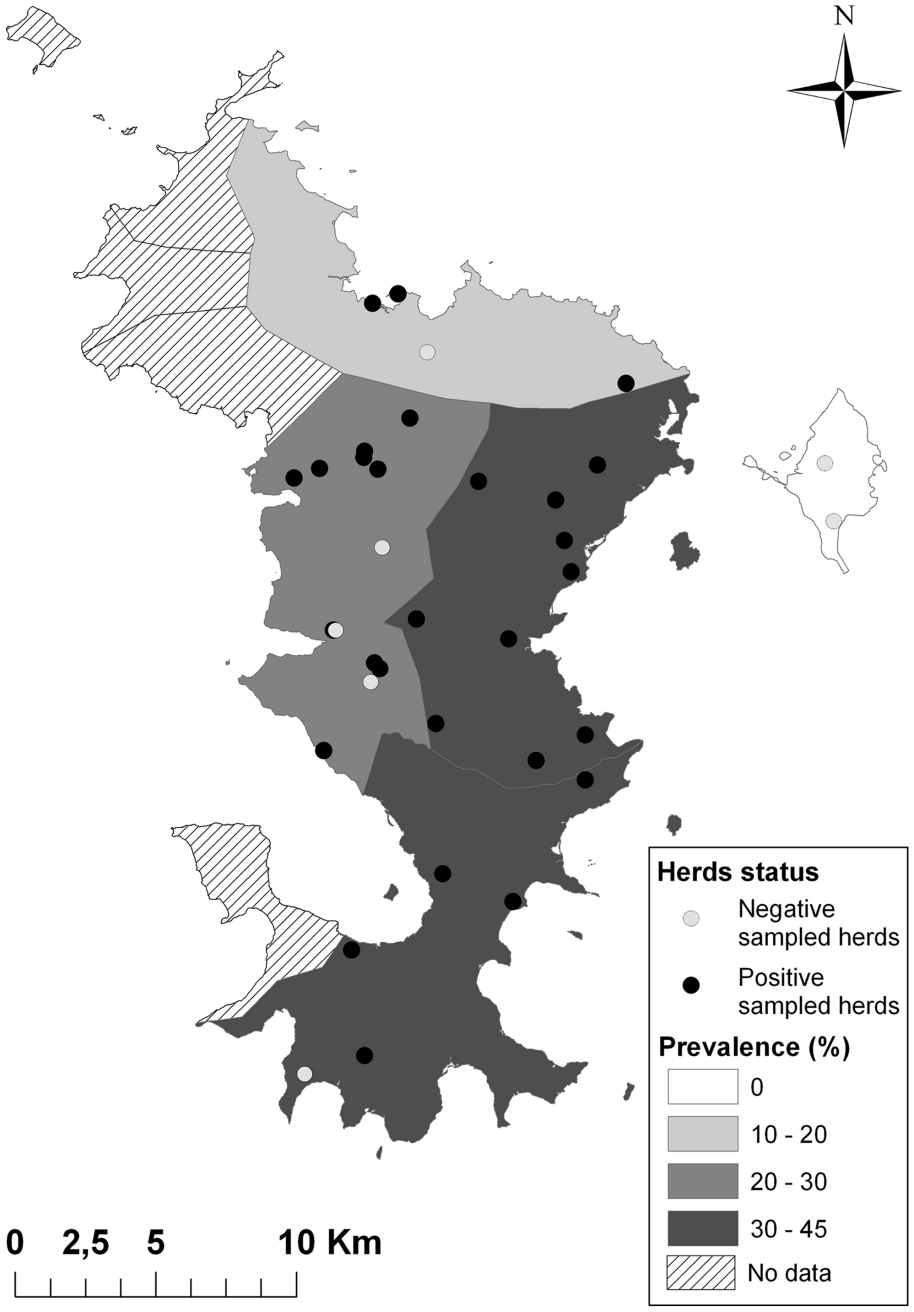
Geographical distribution of ruminant herds included in the study and overall seroprevalence of RVFV IgG antibodies in the ruminant population, Mayotte, 2011.

For the animal study, only ruminant herds were included. Thirty-three sentinel ruminant herds were randomly chosen in different environments and followed up for more than one year, from March 2010 to August 2011; only seronegative ruminants (more than 10 months old for bovines and 6 month-old for small ruminants) at the first visit were included in the follow up (Figure 1). These 198 ruminants (131 bovines and 67 small ruminants; 14 sheep and 53 goats) were blood-sampled each month and each clinical case was reported and further analysed. All the ruminants of these herds (452 adults and young; 378 cattle, 14 sheep and 60 goats) were blood-sampled during the last visit in July 2011.

### Data collection

After enrolment of human participants and blood sampling by a nurse, a questionnaire was administered to each individual to collect socio-demographic data (gender, age, place of birth, schooling, travel history), exposition to potential risk factors (occupation, presence of mosquitoes, contact with animals or blood and organs) as well as protective measures taken. A second questionnaire was administered to the head of the family with questions on housing conditions including composition of the household, building materials and equipment of the house and presence of animals.

For animals, a questionnaire was systematically submitted to cattle owners at the first visit. Information related to the farm characteristics and practices (number of animals, age, housing, feeding…) and the local environment (vegetation type, minimum distance from the farm to the nearest water point, type of the nearest water point, minimum distance from the farm to the nearest forest boundary…) was collected.

### Laboratory methods

The laboratory measure of RVFV exposure in humans was seroconversion, indicated by serum specific IgG RVFV antibodies as marker of past infection, using an in-house indirect ELISA test prepared by the National Reference Centre for Arboviruses (NRC-Arbo) in 2011 at the Institut Pasteur. The NRC-Arbo confirmed the seropositivity of tested samples using in-house MAC and GAG ELISA tests for the capture of human RVFV IgM or IgG, respectively.

The animal serums were analysed using commercial ELISA kits to detect IgG RVFV antibodies (BDSL or IdVet (innovative diagnostics VET, ID Screen® Rift Valley fever Competition Multi-species ELISA)).

### Statistical analysis

Statistical analysis was used to describe the distribution of RVFV-specific IgG positivity among the sampled population (humans and animals). Odds ratios (OR) and their 95% confidence intervals were calculated for exposure factors. For humans, crude prevalence estimations and OR were adjusted according to the inclusion probability of each individual and further weighted by post-stratification for age and gender distribution in the general population (2007 population census). A backwards approach (logistic regression) was used to construct a multivariate model which included risk factors with significance level of ≤0.20 in the univariate analysis. P values <0.05 were considered statistically significant. Weighted results only are presented. For analysis in animals, the contribution of each factor to the model was tested with a likelihood-ratio *χ*2 through a stepwise procedure (backward and forward). At the same time, the simpler models were compared to the full model by the Akaike information criterion [9]. This process was continued automatically until a model was obtained with all factors significant at *P<0.05* (two-sided). Goodness-of-fit of the final model was assessed using Pearson *χ*2 and Deviance tests [10].

We used EPIDATA 3.0 software (Epidata Association, Odense, Denmark) for double data entry and STATA 11.0 (Statcorp. College Station, TX, USA) for statistical analysis.

### Ethics statement

Participation in the human survey was proposed on a voluntary basis. After oral information of participants in the local languages (French, Shimaore, Shibushi), written consent was obtained from adults or guardians of children <18 years prior to the inclusion. For illiterate participants, verbal consent was obtained after lecture of the informed consent letter, with signature of the consent form by an adult family member. All data were collected anonymously. Laboratory results were linked to the questionnaires based on a unique code. The study protocol, including informed consent procedure, was approved by the French competent authorities and ethical committee, in compliance with all French regulations on protection of human subjects (Commission nationale de l′informatique et des libertés (CNIL), n° 910467; Comité de protection des personnes Sud-Ouest et Outre-Mer III (CPP), n° 2010-A00593-36).

Although not compulsory, the study protocol on animals was approved by the ethical committee of Cyroi (Cyclotron Réunion Océan Indien), a technical platform for bio-science in Reunion Island, France. The permission for inclusion of the herds in the study was obtained from each owner on the site. Blood samples were obtained from jugular vein without suffering and were collected in sterile Vacutainer®.

## Results

### Characteristics of participants and seroprevalence in humans

A total of 60 districts, distributed over 43 villages geographically spread on the island of Mayotte were sampled. Out of the 910 households randomly selected to participate, 729 (80%) were enrolled in the study; for 10.5% of households, inhabitants were absent and 9.5% refused participation. The total number of individuals enrolled was 1420, of whom 337 (23.7%) were children aged 5 to 14 years. Refusal of participation of individuals within participating households was higher for children (18%) than for adults (6%). The main reason for refusal was the fear of needles. The mean number of households and individuals included in each district was 13 (range 8–20) and 24 (range 18–26) respectively.

Participants were aged 5 to 90 years, with a mean age of 31 for men and 32 for women. Compared to the whole population (2007 Census), women were over-represented (61.7% versus 51%), as is usually observed in epidemiological studies. Despite inclusion of one child per household, children aged 5 to 14 years were under-represented (23.8% versus 32.8%). Crude data were therefore weighted for age and gender.

Of the 1413 sera that could be analysed, 58 (4.1%) were positive for RVFV IgG. The overall weighted seroprevalence of RVFV antibodies in the general population aged ≥5 years was 3.5% (95% CI 2.6–4.8). The age specific seroprevalence of RVF was very low in children 5–14 years old and was highest in the age group of 15 to 34 years, with a decrease in older age groups (Table 1). No significant difference was observed according to gender and geographical area. The highest seroprevalence rates were observed in farmers (32.1%) and persons with animal birthing activity or in contact with aborted animal fetuses (27.8%).

**Table 1:** Adjusted prevalence of RVFV IgG antibodies in the general population ≥5 years and Odds ratios in univariate analysis according to demographic variables and potential risk factors, Mayotte, 2011.

Variable	Subcategory	Tested individuals (N)	Adjusted prevalence % (95% CI)	Adjusted OR (95% CI)	p
Gender	Male	542	4.3 (2.7–7.0)	1	
	Female	871	3.0 (2.1–4.3)	0.69 (0.39–1.23)	0.20
Age (years)	5–14	335	0.4 (0.8–1.7)	1	
	15–34	495	5.4 (3.7–8.0)	15.21 (2.97–77.78)	0.001
	35–54	410	4.6 (2.5–8.1)	12.70 (2.51–64.16)	0.003
	≥55	168	2.8 (1.1–6.7)	7.70 (1.60–36.80)	0.01
Birthplace	Mayotte	806	1.4 (0.6–3.0)	1	
	Comoros	528	7.7 (5.5–10.8)	6.10 (2.43–15.37)	0.000
	Other	63	0	-	
Residence in Mayotte	<5 years	155	10.0 (5.1–18.8)	1	
	≥5 years	1248	2.9 (2.0–4.1)	2.78 (1.37–5.34)	0.001
Educational level	Primary school or less	817	4.5 (3.2–6.4)	1	
	College up to university	588	1.8 (1.0–3.3)	0.37 (0.17–0.71)	0.002
Geographical area	North	215	3.2 (1.4–6.9)	1	
	Center	261	4.1 (3.0–5.4)	1.29 (0.54–3.08)	0.56
	Mamoudzou capital town	478	3.0 (1.7–5.3)	0.96 (0.35–2.62)	0.93
	Petite Terre	172	2.2 (0.8–5.6)	0.68 (0.19–2.45)	0.55
	South	287	4.9 (2.3–10.1)	1.57 (0.51–4.87)	0.43
Occupation	Administrative	761	1.3 (0.7–2.4)	1	
	Farmer	19	32.1 (13.4–59.0)	35.21 (9.36–132.35)	0.000
	Unemployed	633	5.5 (3.9–7.7)	4.34 (2.20–8.55)	0.000
Slaughtering	No	1289	3.3 (2.3–4.5)	1	
	Yes	124	6.7 (2.8–15.1)	2.15 (0.81–5.70)	0.12
Birthing or abortion	No	1405	3.3 (2.5–4.5)	1	
	Yes	8	27.8 (3.8–78.8)	11.12 (1.10–112.3)	0.04
Handling dead animals	No	870	3.3 (2.1–5.1)	1	
	Yes	543	3.8 (2.4–6.1)	1.16 (0.57–2.35)	0.68
Cooking fresh meat	No	730	3.0 (1.9–4.7)	1	
	Yes	386	4.2 (2.8–6.1)	1.41 (0.77–2.55)	0.26
Raw milk consumption	No	885	3.8 (2.5–5.7)	1	
	Yes	528	3.2 (1.9–5.3)	0.88 (0.45–1.72)	0.70
Fresh blood consumption	No	1408	3.5 (2.6–4.8)	1	
	Yes	5	0	0 (0–18.12)	0.93
Environment	Urban	751	2.9 (1.7–4.7)	1	
	Rural	661	4.2 (2.8–6.2)	1.47 (0.74–2.93)	0.65
Nearby water source	No	628	2.0 (1.1–3.6)	1	
	Yes	784	4.8 (3.3–7.0)	2.46 (1.17–5.16)	0.02

### Seroprevalence and incidence in animals

A total of 33 farms (198 ruminants), spread on the island of Mayotte, have been regularly sampled (Figure 1). The overall seroprevalence of RVFV antibodies in the ruminant population was 25.3% (95% CI 19.8–32.2) in August 2011 but 61.9% of the farms (95% CI 44.8–79.2) were positive. No significant difference was observed between small ruminants (22.4%–5% CI 14.1–32.2) and cattle (26.8%–5% CI 19.1–35.3). The animal incidence from the 1^st^ quarter 2010 to the 3^rd^ quarter 2011 was 12.9% (95% CI 9.6–16.2) with a peak of 18.8% for the 4^th^ quarter 2010, during the rainy season.

### Analysis of risk factors for humans

In univariate statistical analyses, RVFV seropositivity varied significantly according to the following factors: age (≥15 years of age), place of birth (higher risk for people born on the Comoros), duration of residence in Mayotte (increased risk if <5 years), low educational level, professional activity (higher risk for farmers and unemployed people), assist with animal birthing or disposal of aborted animal fetuses, and living close to a water source (river, swamp, water draining system…) (Table 1). Gender, environment, activities of slaughtering, cooking fresh meat, handling dead animals, and consumption of raw milk or fresh blood were not significantly associated with an increased risk of RVF in univariate analysis (Table 1). Other studied factors that did not significantly influence RVF seroprevalence were: history of regular travel to countries where outbreaks of RVF occurred, protection against mosquitoes and housing conditions (access to piped water, type and size of the house and number of inhabitants) (data not shown).

Multivariate analysis identified the following factors to be significantly associated with RVFV seropositivity: age (≥15), gender (men), place of birth on the Comoros, living in Mayotte since less than 5 years, low educational level, farming and living close to a water source (Table 2).

**Table 2:** Final logistic regression model presenting significantly associated risk factors for RVF in the general population ≥5 years, Mayotte, 2011.

Variable	Subcategory	Adjusted OR (95% CI)	p
Gender	Male	2.33 (1.19–4.55)	0.01
	Female*	1	
Age	5–14*	1	
	≥15	6.17 (1.11–34.29)	0.04
Birthplace	Mayotte/other*	1	
	Comoros	3.28 (1.43–7.45)	0.006
Residence in Mayotte	<5 years	2.96 (1.39–6.31)	0.006
	≥5 years*	1	
Educational level	Primary school or less	2.34 (1.04–5.23)	0.04
	College up to university*	1	
Occupation	Administrative*	1	
	Farmer	9.24 (1.50–56.81)	0.02
Nearby water source	No*	1	
	Yes	2.24 (1.03–4.88)	0.04

* reference category.

### Analysis of risk factors for ruminants

In univariate statistical analysis, significant risk factors for RVFV seropositivity were: new animals introduced in the herd (lent from other farms), selling of animals, no use of insecticides on cattle or deworming treatment (with albendazole), farm located nearby a water point (≤1000 m), no constant access to a water point, no immediate disposal of aborted fetuses, and heavy rainfall (>180 mm) in the previous 2 months (Table 3). Animal feeding conditions, keeping cattle in the farm with or without small ruminants, a daily route for cattle in the forest, fighting against rodents, presence of ticks, type of vegetation in which ruminants are grazing or potential contact with other ruminants were not significantly associated with an increased risk of RVF in univariate analysis (data not shown).

**Table 3:** Adjusted prevalence of RVFV IgG antibodies in the ruminant population and Odds ratios in univariate analysis according to potential risk factors, Mayotte, 2011.

Variable	Subcategory	Tested individuals (N)	Adjusted prevalence % (95% CI)	Adjusted OR (95% CI)	p
Type of production	Milk and meat	253	33.6 (29.1–38.1)	1	
	Milk or meat	169	35.5 (30.9–40.1)	1.10 (0.75–1.65)	0.59
Selling of animals	No	52	17.3 (13.7–20.9)	1	
	Yes	370	36.7 (32.1–41.3)	2.70 (1.29–5.72)	0.008
Buying of animals	No	260	34.2 (29.6–38.8)	1	
	Yes	162	34.5 (29.9–39.1)	1.11 (0.67–1.52)	0.95
Reception of lent animals*	No	148	26.3 (22.1–30.5)	1	
	Yes	274	38.6 (34.0–43.2)	1.79 (1.16–2.78)	0.009
Lending of animals	No	381	34.1 (29.6–38.6)	1	
	Yes	41	36.5 (31.9–41.1)	1.13 (0.56–2.13)	0.78
Management of dead animals	Good process	157	33.6 (29.1–38.1)	1	
	Bad process	265	35.5 (30.9–40.1)	1.10 (0.66–1.35)	0.6
Management of dead fetuses*	Eliminated	223	30.0 (25.6–34.4)	1	
	Kept into the farm	199	39.1 (34.5–43.7)	1.47 (1.13–2.17)	0.04
Use of insecticides*	Yes	210	27.1 (22.9–31.3)	1	
	No	212	41.5 (36.9–46.1)	1.82 (1.30–2.86)	0.005
Use of acaricides	Yes	360	33.8 (29.3–38.3)	1	
	No	62	37.0 (32.4–41.6)	1.10 (0.67–1.16)	0.75
Deworming*	Yes	98	26.5 (22.3–30.7)	1	
	No	324	36.7 (32.1–41.3)	1.65 (1.15–2.63)	0.04
Nearby water point*	No	208	22.5 (18.5–26.5)	1	
	Yes	214	45.8 (41.0–50.6)	1.55 (1.13–2.12)	0.007
Constant access to water point*	Yes	98	26.5 (22.5–30.5)	1	
	No	324	36.7 (31.2–41.3)	1.62 (1.13–2.12)	0.04
Distance to the water point*	>1000m	202	28.2 (24.2–32.2)	1	
	≤1000m	220	40.1 (35.4–44.8)	1.72 (1.15–2.56)	0.01
Previous two month rainfalls*	≤180mm	227	25.1 (21.1–29.1)	1	
	>180mm	195	45.1 (40.3–49.9)	2.56 (1.67–3.85)	0.0001

* variable retained after univariate analysis for the logistic model.

Multivariate analysis identified only 3 major risk factors to be significantly associated with RVFV seropositivity: location of the farm nearby a water point, heavy rainfall in the previous two months and absence of abortions disposal (Table 4).

**Table 4:** Final logistic regression model presenting significantly associated risk factors for RVF in the ruminant population, Mayotte, 2011.

Variable	Subcategory	Adjusted OR (95% CI)	p
Nearby water point	No*	1	
	Yes (permanent or not)	1.55 (1.13–2.12)	0.04
Previous two month rainfalls	≤180mm*	1	
	>180mm	2.22 (1.45–3.45)	0.003
Management of dead fetuses	Eliminated	1	
	Kept into the farm*	1.82 (1.12–2.94)	0.01

* reference category.

## Discussion

The seroprevalence of Rift Valley Fever virus IgG antibodies in the general population aged 5 years or more in Mayotte estimated by this first large scaled study was 3.5%, with the highest prevalence observed in the 15 to 34 years old (5.4%). Because in-house indirect ELISA has been used for the detection of RVF antibodies in tested sera, all dubious and positive biological samples were subsequently validated by Mac and Gag ELISA tests with a high degree of confidence. Such serological methods were particularly suitable for limiting the risk of a biased estimate of the prevalence rate. During outbreaks, prevalence rates up to 32% have been registered [11], but interepidemic seropositivity in different contexts and countries varied between 3% in Tanzania, 13% in Kenya and 22% in Senegal [12]–[14]. In a rural population in Gabon, a country where like Mayotte, no outbreaks of RVF have ever been reported, seroprevalence was estimated at 3.3% [15].

The seroprevalence of Rift Valley Fever virus IgG antibodies in ruminants older than ten months, estimated by this follow up was 25.3%, much higher than the one observed in humans. These results are in accordance with those from previous studies in Mayotte conducted from 2004 to 2007, where prevalence rates between 12 and 32% were registered [6]. They are also similar with observations from the other islands in the Indian Ocean, in Comoros [16] and in Madagascar [17], where the seroprevalence rates were 32.8% in 2009 and 25.6% in 2008, respectively. These data were obtained from livestock during inter-epizootic periods, but the rates observed are close to those reported during or close after epizootics, for example in Kenya [18] or in Mauritania [19], where prevalence rates from 12% to 33% were registered. However seroprevalence results in animal studies need to be balanced regarding the composition of herds. Indeed, goats are highly susceptible to infection, even if they appear to be more refractory to severe or lethal disease than sheep and cattle. Several studies revealed that approximately 2% to 10% of goats are seropositive for anti–RVF virus antibody during enzootic periods but this percentage rose up to 70% following epizootics [20], [21]. In our study, we sampled bovines and small ruminants, mainly goats. However, no significant difference of prevalence was observed between cattle and small ruminants. The incidence rate obtained from the follow up of animal herds was 12.9%, much higher than the ones observed in Senegal (5.4%) in small ruminants or in Kenya (4.9%) in livestock [21], [18].

Both the studies in humans and in animals indicate that the RVFV has been circulating actively on the territory of Mayotte, since at least 2004. Several arguments tend to indicate that the virus is probably imported from nearby Comoros islands, affected by RVF because of regular imports of live ruminants from eastern Africa, mainly Tanzania [16]. The human cases of RVF diagnosed in Mayotte in 2007 and 2008 were genetically linked to the Kenyan outbreak of 2006–2007, that also affected the Comoros [22]. In our study, people born in the Comoros or those recently living in Mayotte (<5 years) are at greater risk of RFV infection than people born in Mayotte (see further). And high seropositivity rates (37%) have been observed among animals illegally imported from the Comoros islands (Anjouan mainly) and tested at their arrival, suggesting that illegal animal movements are a likely source of RVFV introduction in Mayotte [6].

However, seroprevalence rates obtained in animals from 2004 to 2011 and the high incidence rate in 2010–2011 in ruminants strongly support the evidence of maintenance of the virus circulation in Mayotte, in addition to regular import. A peak of 18.8% incidence was observed in 2010 during the rainy season, a favorable period for mosquitoes to multiply. During periods with less-excessive rainfalls, RVF virus is likely maintained in an enzootic cycle within the mosquito vector population, involving transovarial transmission with occasional infection and amplification of virus in susceptible cattle or small ruminants [23]. Several mosquito species that have been identified as possible vector for RVF are present in Mayotte, including *Ae. circumluteolus*, *Cx.antennatus*, *Cx. quinquefasciatus* and *Ae. Aegypti* [6], [24]. In 2009, the virus was found (by PCR) on a *Ae. simpsonii* mosquito [25]. And a recent inventory of mosquitos captured around the animal herds included in our study described 40 mosquito species belonging to 9 genera, including all the competent species for RVFV transmission [26] suggesting that the virus could be easily transmitted to livestock by these insects.

Age ≥15 years has been identified as a possible risk factor for RVF seropositivity in humans. However, the low seroprevalence in children 5 to 14 years old is probably related to behaviour factors and less cumulative exposition to mosquitoes and does not indicate that the virus circulated more on the island 15 years ago.

As found in other studies [13], [15], men living in Mayotte were three times more exposed to RVFV than women. A possible reason is that men spend more time outside the house and are thus more exposed to mosquito bites.

The highest association to RVFV seropositivity (in univariate analysis only, small number) was found with assisting animals during birth or abortions (OR of 27.8), also described in Senegal and Kenya [13], [14]. Slaughtering, cooking fresh meat, contact with dead animals and drinking raw milk were not significantly associated with RVF infection.

In our study, people with a low educational level (no schooling or primary level only) were at greater risk for RVFV infection than those with a higher level.

The higher exposure of people born in the Comoros and recently living in Mayotte (<5 years) suggest that RVFV circulation was more important in the other islands of the Comoros archipelago than in Mayotte. However, Comorian people might have been more exposed to the virus after their arrival on Mayotte than local people, through occupational activities such as farming, found to be a main risk factor for RVFV infection (OR 9.2). Indeed, farms often belong to Mahorais people but daily work is carried out by immigrants. Travelling to the Comoros islands is not significantly associated to a higher RVFV seropositivity.

Proximity to a water source, identified as a risk factor in Mayotte was also described in Tanzania, Gabon and Saudi Arabia [12], [15], [27]. Although protection against mosquito bites did not significantly influence on RVFV seropositivity, it seems that mosquitoes and other bloodsucking vectors play an important role in RVFV transmission on the island.

As for humans, a main risk factor associated with animal RVFV seropositivity was the presence of a nearby water point from the farm or the night-pen for ruminants animal, confirming that a substantial part of the virus transmission in Mayotte could be carried out by mosquito vectors. Most of the mosquito species identified as potential vectors for RFV in Mayotte [26], particularly Culex and Aedes genera, have a main crepuscular and nocturnal activity [28], and given that cattle come back to the farm in the afternoon, a great distance between animals and the water point limits the risk to be bitten by mosquitoes and then the risk to be infected. The abundance of the rainfalls is a crucial factor for the setting of mosquitoes breeding sites and low rainfall (less than 180 mm per month) especially during the rainy season was associated with a lower animal seropositivity. Indeed, persistent rainfall creates non-permanent water points, resulting in new breeding sites for mosquitoes.

Another risk factor for ruminants associated with a higher seropositivity was non-disposal of aborted fetuses. A systematic elimination of dead fetuses after abortions (dead fetuses could be buried, burnt or consumed) reduced the direct risk of virus transmission; as for humans, ruminants can become infected through contacts with blood, tissues, secretions or excretions of infected animals, notably after abortion [29]. Aborted fetal materials and placental membranes contain large numbers of virus particles which can either contaminate the local environment directly or infect animals in close contact. The RVFV may persist for relatively long periods in the environment as it has been demonstrated during in vitro experiments [30].

Although the human and animal serosurveys suggest significant circulation of RVFV on the island, very few human clinical cases have been detected and few abortions or mortality among young ruminants reported, despite active and passive surveillance. Infection in humans is often asymptomatic and clinical signs, when present, are non-specific for RVF. However, in 2010 and 2011, more than 1400 PCR-tests for RVFV were performed in Mayotte, on people presenting with fever and dengue-like illness. All were negative. The absence of clinical cases in animals might be explained by a sufficiently high density of susceptible animals on the island to sustain virus circulation but not high enough to support waves of epidemic abortion and death [6]. Before any launching of epizootics, multiple infected mosquito bites are required to generate a high level of viremia in animals and only when considerable number of ruminants are heavily infected, amplification could take place through direct transmission of the virus with infected tissues and propagation with blood-sucking insects [23]. During these inter-epizootic periods, viremia in ruminants remain low resulting in few infected mosquitoes and an absence of symptomatic cases in animals and humans. Alternatively, the RVFV virus may persist in an unidentified vertebrate reservoir [31], but our first studies carried out in 2011 on 100 rats and 50 lemurs, potential wild reservoirs of the virus, revealed no presence of specific antibodies (unpublished data).

## Conclusions

Although resulting in few clinical cases in humans and in animals, RVFV has been circulating actively on the island of Mayotte since several years, in a context of regular import of the virus from nearby countries through illegal animal movements, the presence of sufficient numbers of susceptible animals and a favorable environment for mosquito vectors to maintain virus transmission locally.

Several risk factors highlighted that humans and animals share the same ways of RVFV transmission. Non disposal of an aborted animal fetus as risk factor may indicate the importance of virus transmission by aerosolization of blood and amniotic fluid during animal birthing. Exposure to infected tissues and body fluids remains the main route of infection for humans but is also at risk for ruminants [32]. Proximity to a water source confirmed that abundant mosquito breeding places and then abundant mosquito population could facilitate transmission to ruminants and to humans [1].

Finally, farming and cattle ownership are important factors for RVFV seropositivity; such a result was expected since ruminants are the main animal host of RVFV.

Our studies emphasize the need for a one health approach in which humans and animals within their ecosystems are included. Only these holistic approaches that acknowledge the interdependence of people, domestic and wild animals, and the environment will better address today's dynamic health threats and propose adequate control measures to limit these threats.
